# Engineering-based statistical analysis of amputation prevalence and the impact of contributing factors in 4 Iraqi cities (1980–2024)

**DOI:** 10.1016/j.cjtee.2025.06.003

**Published:** 2026-03-24

**Authors:** Samer A. Kokz, Amer Matrood Imran, Adnan K. Shathir

**Affiliations:** Prosthetics and Orthotics Engineer Department, College of Engineering, University of Kerbala, Iraq

**Keywords:** Prevalence of amputees, Iraqi amputees, Cause of amputation, Level of amputation

## Abstract

**Purpose:**

The number of amputations, which rises during specific periods in most countries, is due to numerous causes and is accompanied by continuous psychological, social, and economic effects for patients. This study aims to construct a data bank of amputees for the first time by collecting and analyzing data from 4 health centres in Iraq (the health centres of the amputee in Karbala, Najaf, Babylon, and Wasit cities).

**Methods:**

This study was conducted retrospectively on all amputees in dataset for the period from January 1980 to December 2024. The inclusion criteria for the data bank include clinical and demographic data for amputation cases, such as sex, age, ID, type of amputation, date of amputation, cause of amputation, level of amputation, name of health center, etc. Amputation cases were excluded from this study that had missing data, unclear records, outside the chosen cities, and redundant or repeated records.

**Results:**

There were 9030 subjects (7446 male and 1584 female) included in this research. The results show that the highest rates of amputation were noticed in (40–69 years) for the age groups. Furthermore, it significantly indicated that females had a lower amputation rate compared to males. Also, the largest percentage of amputation was in Babylon followed by Najaf, Karbala, and Waist among the 4 selected cities. The major reason for amputation was the wars in Iraq. The below-knee amputation was the most common. Additionally, most of the decades that occurred amputations were in the years 2010–2019 that followed 1980–1989. We also noted that Iraq has the largest number of amputees compared to the other countries included in the investigation.

**Conclusion:**

Based on these results, future potentials must focus on planting a national Iraqi database for amputees every year. Also, it should find the premature diagnosis of all diseases that cause amputations. All these processes can be performed through the cooperation between health centres, research centres in the universities, and the government of Iraq.

## Highlights

• Creation of Iraq's first comprehensive amputee data bank. Collection of Information from four centres (Karbala, Najaf, Babylon, and Wasit) start January 1980 to December 2023.
• Amputation is caused by wars, conflicts, health conditions, diabetes, and accidents for 9030 Iraqi individuals (7446 males and 1584 females).
• Iraq shows the highest number of amputees among the countries studied. Significant increases in amputations during 1980–1989 and 2010–2019.
• Most amputees are aged 40 and above. Below-knee amputations are the most common.

## 1 Introduction

Amputation is a major health and medical matter worldwide. The number of amputees is rising continuously due to medical problems such as vascular diseases, diabetes, and cancerous tumors. Also, the injuries and trauma (like falls, traffic accidents, industrial accidents, severe burns, and frostbite), and congenital malformations are increasing constantly.^1^ Iraq suffered for decades of war, landmines, armed conflicts, traffic accidents, and chronic diseases that led to a significant increase in the number of amputees. It has become necessary to collect the facts and data about the number of amputations, the amputation status, the amputation type, the distribution of amputees in cities, and their response to treatment by using prosthetics or wheelchairs.^2^ Despite the rate of amputation being relatively high, unfortunately, no precise data describes the distribution and causes across major Iraqi cities. Hence, this research aims to analyze the major and clear cases of amputation in 4 main cities in Iraq. These accurate data and statistics assist research and medical centres that are interested in amputation issues by suggesting, implementing, and applying the best plans.^3^

The rate of amputation has varied across countries, with a study in Germany revealing a 25.4% increase in minor amputations compared to a 30.9% decrease in major amputations.^4^ The World Health Organization identifies road traffic accidents as a significant global issue, resulting in many amputations.^5^^,^^6^ Diabetes is a risk factor that affected the amputation rates significantly. According to a study, the amputation rates in both the 100 traumatic admissions and the 100,000 deployed troops have notably increased.^7^ Notably, 30% of amputees had undergone multiple amputations for the upper and lower extremities. Additionally, 228 amputations affected the upper extremities, accounting for 14% of the total. Furthermore, an analysis of lower extremity amputation rates across 10 global centres revealed that the Navajo population (native American community in the United States) had the highest rates, with 43.9 amputations per 100,000 males annually.^8^ Madrid, Spain, had the lowest rates, with just 2.8 amputations per 100,000 people. In Germany, a population-based study found a 11.1% decrease in lower limb amputations (LLAs) from 2005 to 2015, with higher amputation levels experiencing the greatest declines. Sex was found to have a direct association with amputation rates, with males having a higher incidence. Diabetes was found to be a contributing factor in 25%–90% of amputations.^8^ In-hospital mortality fell from 19.8% to 17.4%, and reamputation rates decreased from 13.2% to 10.2%.^9^ A systematic review of major lower extremity amputations in the United Kingdom found a range of 8.2 to 51.1 per 100,000 individuals in the general population and 70 to 291 per 100,000 individuals in the diabetic community. There was no definitive indication of a rising occurrence, with higher incidence consistently found in northern England. Variations in major lower extremity amputation incidence were attributed to differences in database use, definitions, calculation methods, and procedure inclusions.^10^ The increase in major amputation rates from 1979 to 1995 was 10.6%, despite a decline from 1983 to 1991. The decline was correlated with reduced smoking, hypertension, and heart disease, but not diabetes. Amputation and angioplasty rates were inversely correlated in the 1980s. The major amputation rate increased post-1993, reaching 24.95 per 100,000 in 1996.^11^

A study comparing LLAs in Japan and Australia found that adults with diabetes have a significantly higher rate of both major and minor LLAs.^12^ Diabetes-related foot disease (DFD) is a significant contributor to hospitalisation, amputation, and impairment worldwide. Additionally, it determines the prevalence and incidence of risk factors, ulcers, infections, and amputations associated with DFD in Australian populations. Twenty articles met the inclusion criteria, showing significant variation in populations and methodologies used. The prevalence of risk factors varied from 10.0% to 58.8%, while the prevalence of DFD ranged from 1.2% to 1.5%. The incidence of diabetes-related amputations ranged from 5.2 to 7.2 per 1000 person-years.^13^ The Karolinska University Hospital study found a 60% reduction in above-ankle amputations among patients with diabetes, but found higher incidences of foot infections and renal diseases. Females with diabetes were older at amputation procedures, while males had a higher frequency of multiple amputations and infections. Additionally, foot ulcers caused 88% of diabetes-related amputations, with only 30% referred to multidisciplinary foot teams.^14^

In Germany, a study investigated the impact of cardiovascular risk factors, comorbidities, and in-hospital mortality on individuals undergoing LLAs due to peripheral arterial disease (PAD) or critical limb ischemia. Results showed a decrease in major LLAs by 21.8% in 2009, while minor LLA rates increased by 2%. Revascularization rates increased from 46% in 2005 to 57% in 2009, but the overall number of ischemia-related LLAs and mortality rates remain high.^15^ Additionally, 60 individuals involved in LLAs due to peripheral vascular disease found that 78.8% experienced phantom limb pain (PLP), while 51.2% experienced stump discomfort and 15.4% experienced unusual phenomena. The researchers found significant correlations between PLP and stump discomfort and the ability to regulate phantom limb movement, but no significant correlation between PLP and contraction of the phantom limb. The study highlights the importance of understanding and managing phantom sensations in amputation recovery.^16^ The prevalence of PLP in individuals with LLAs was analyzed for 798 participants, of which 79% had phantom limb symptoms, 72% experienced pain, and 74% experienced residual limb discomfort. The study found that pain-related functional limitations were relatively low, with a significant proportion of patients experiencing difficulty in other areas, such as the back. After 6 months and 1.5 years, phantom pain was most common in women, and the percentage of prosthetic use dropped to 60%. The study found that LLA, male gender, and longer post-amputation time protect against phantom pain.^17^

By looking at amputees’ age, amputation degree, and amputation cause, which affected anxiety and depression in people who had 1 lower limb amputated, we found that the transfemoral amputation group had significantly higher Hospital Anxiety and Depression Scale-A scores than the transtibial amputation group. Patients aged ≤35 had higher K index values.^18^ Moreover, research from 2004 to 2016 revealed that 89% of amputees were male, with the 20–29 years age group experiencing the highest incidence. Most injuries occurred at the workplace, with electrical burns and hot crush injuries being the most common causes, of which 60.4% achieved primary healing. From 2007 to 2009, a study in Scotland investigated the impact of gender, amputation level, and diabetes mellitus on prosthetic limb fitting following a LLA due to PAD. Results showed that younger age and transtibial amputation were predictors of prosthetic limb fitting, but success rates were similar between those with and without diabetes.^19^

Researchers used accelerometers and questionnaires to assess the impact of postoperative prostheses on patients' activity levels and quality of life after transtibial amputation. The study involved 10 patients, mostly amputated due to gangrenous ulcers. Over a 6-week follow-up, patients spent 88% of their time sedentary, with only 0.3% engaging in moderate to vigorous activity. Despite the prosthesis, physical and emotional Short Form-36 scores were low, indicating high sedentary behavior and low quality of life. The study also evaluated health-related quality of life and prosthesis use in 98 patients with major amputations due to PAD.^20^ A study assessed 30 patients before and after prosthetic fitting using various tests, finding significant improvements in EuroQol-5 Dimensions, Prosthetic Limb Users Survey of Mobility, and Two Minutes Walking Test post-prosthesis.^21^ The results were more pronounced in non-vascular amputees, with 84% of non-vascular and 44% of vascular amputees using their prostheses within 1 year. Prosthesis provision significantly improves quality of life and function, particularly in non-vascular cases. Ten patients with transtibial amputations showed improvements in their physical activity levels and overall quality of life after receiving an immediate postoperative prosthesis. However, patients spent 88% of their time sedentary, with no significant correlation between expected function and activity level. The study also assessed rehabilitation outcomes for 17 bilateral lower limb amputees under the StandTall program following the 2008 Sichuan earthquake. Factors associated with successful recovery included amputation level, knee joint salvage, prosthesis use, exercise, and education.^21^

After examining 45 patients with major proximal limb amputations due to sarcoma, we examined their postoperative progress, survival rate, pain levels, quality of life, and prosthetic limb usage. Out of 1597 patients, 2.3% underwent amputation. The survival rate was 42.9%, decreasing to 12.7% after 5 years. Full tumour resections increase the likelihood of survival. Postoperative chemotherapy and radiation did not improve survival, of which 18% experienced local recurrence, 44% reported postoperative problems, and 87% experienced phantom pain. Only 32% of prostheses users used them consistently.^22^ Wound size, classification, white blood cell count, neutrophil percentage, haemoglobin, C-reactive protein, and albumin are all significant risk factors for major amputations. Individuals with major amputations had lower education, poor nutritional status, decreased physical activity, and more advanced wound diseases. Additionally, the findings exhibited elevated white blood cell counts, elevated C-reactive protein levels, and decreased albumin levels.^23^

As is well known, the Middle East, especially Iraq passed through many difficult circumstances, such as the many wars, the spread of cancer, and the prevalence of various accidents. As a result of these reasons, the number of amputations increases directly in almost all Iraqi cities from north to south. The researchers and workers in this field need the Data Information Bank that is a useful reference to the Medical Centres of Amputation Rehabilitation. Therefore, the objective of this study is to analyze the prevalence of amputees and the variations in the causes of amputation in 4 cities in Iraq. This analysis is based on a dataset collected from 4 health centres. The findings of this study could assist specialists in identifying the issues and developing a robust system to support the amputee community in Iraq.

## 2 Methods

The study used the amputee dataset, which gathered data from many Iraqi Health Centres (IHCs) from January 1980 to September 2024. The dataset had a total of 9030 individuals. This flowchart in Fig. 1 illustrates the structured process of collecting, cleaning, categorizing, and statistically analyzing amputee data. The study included the health centres of the amputees in Karbala, Najaf, Babylon, and Wasit cities.

**Fig. 1:**
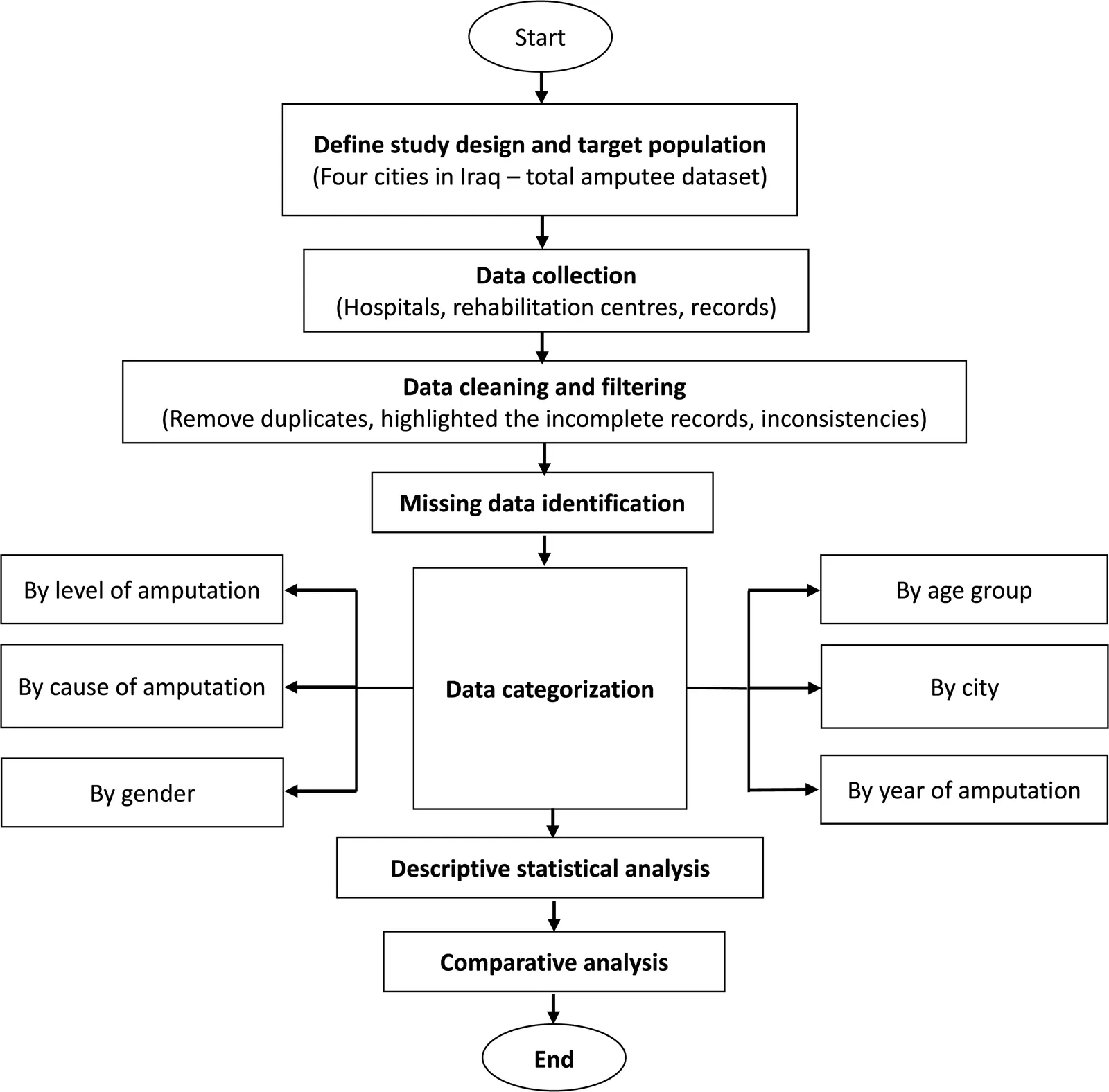
Methodological framework for epidemiological analysis of amputees in 4 Iraqi cities.

### 2.1 Inclusion and exclusion criteria

The 4 health centres were selected because of the extensive visitor base of individuals with amputations and the accuracy of their data-gathering techniques compared to other health centres in Iraq. They recorded each instance of patients requesting treatment, assistance with orthotic and prosthetic devices, or maintenance for prosthetic limbs. The first dataset obtained from centres included all the amputee patients registered in the centres’ 45,000 diverse occurrences, including upper limb amputations, orthosis deviations, and LLAs.

To conduct this study, the dataset was filtered to only include the documented lower limb amputations in the medical centres, yielding a total of 9030 instances, including 7446 males and 1584 females. Additionally, the study removed the duplicated name as registered in more than one centre to maintain the accuracy of study data. The patients of all ages and both sexes were included during the study. The amputee patient with minimum essential variables was also considered during the analysis, trying to prove the importance of data assigned to the healthcare system and patient welfare. This study excluded patient names due to ethical considerations and considered the variables such as age, sex, centre name, level of amputation, and cause of amputation.

The exclusion records were for the amputee patient with an upper amputation, and the names appeared in more than 1 centre, and the patient registered outside of the study period. The study excluded the patients who visited the centres for rehabilitation service and orthotic care and were not amputees. The repeated name of the amputee who was for follow-up visited in the same centre was also excluded.

The participants in this study had surgical interventions to amputate a limb from 1981 to 2014. The extensive duration allows for a comprehensive analysis of several factors that influence different aspects of amputees' lives. The wide age range of patients allowed for a comprehensive investigation into the prevalence and repercussions of amputations across different age groups. The study aimed to analyze each case in terms of age, gender, level of amputation, cause of amputation, and year of amputation to determine the prevalence rate of amputations in 4 Iraqi cities.

### 2.2 Statistical method

The data of the lower amputee patient were entered, cleaned, coded, and analyzed by using Microsoft Excel software and IBM SPSS software. Linear regression models were used to fill in the missing values based on how the other variables were related to each variable. Using a model makes it easier to get reliable values for missing data while keeping bias and statistical power to a minimum. Regarding missing data, records with missing essential variables, such as, age, sex, or amputation status, were analyzed to illustrate their effect on the results, while records with missing secondary variables were analyzed using available-case analysis. The number of excluded records and the reasons for exclusion were documented and reported to reduce bias and improve the transparency and reliability of the study findings. Comparisons between the 4 centres and different countries were performed to show the prevalence rate of Iraq compared to other countries.

## 3 Results

The distinct impact on persons aged 40 to 69 who have undergone amputation, compared to other demographic categories (Fig. 2). The rise may be attributed to both an increased probability of health issues and the consequences of wars and conflicts in Iraq. The findings also indicate that a considerable proportion of individuals with amputation did not provide their age, a critical factor that significantly influences both research validity and the rehabilitation process. The substantial rise in amputation prevalence among the 40–50 and 50–60 age groups is driven by multiple factors, including chronic illnesses, war injuries, diabetes, and traffic accidents, as shown in Fig. 3.

**Fig. 2:**
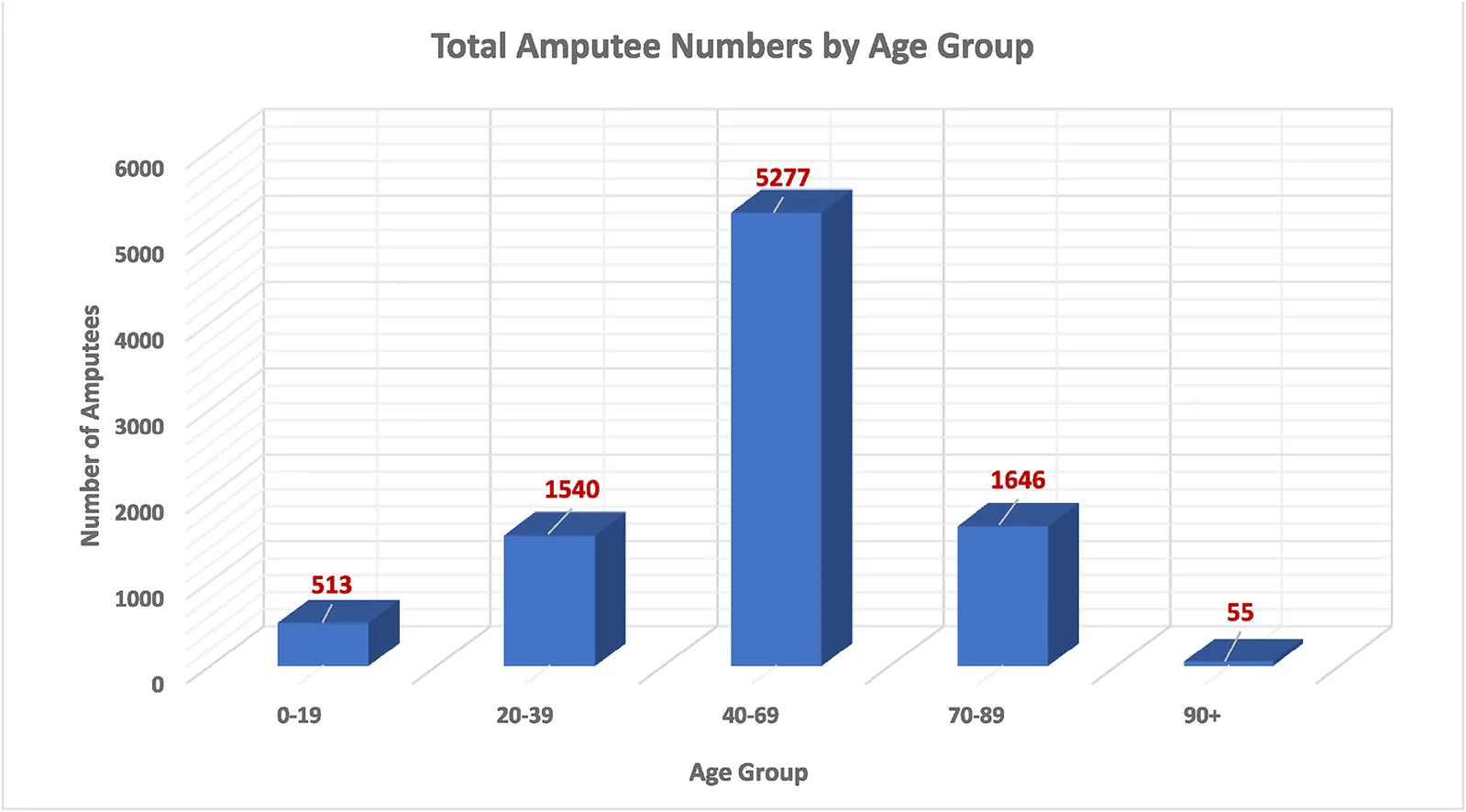
The impact of age on the occurrence of amputation in Iraq, specifically focusing on the prevalence rates across different age groups.

**Fig. 3:**
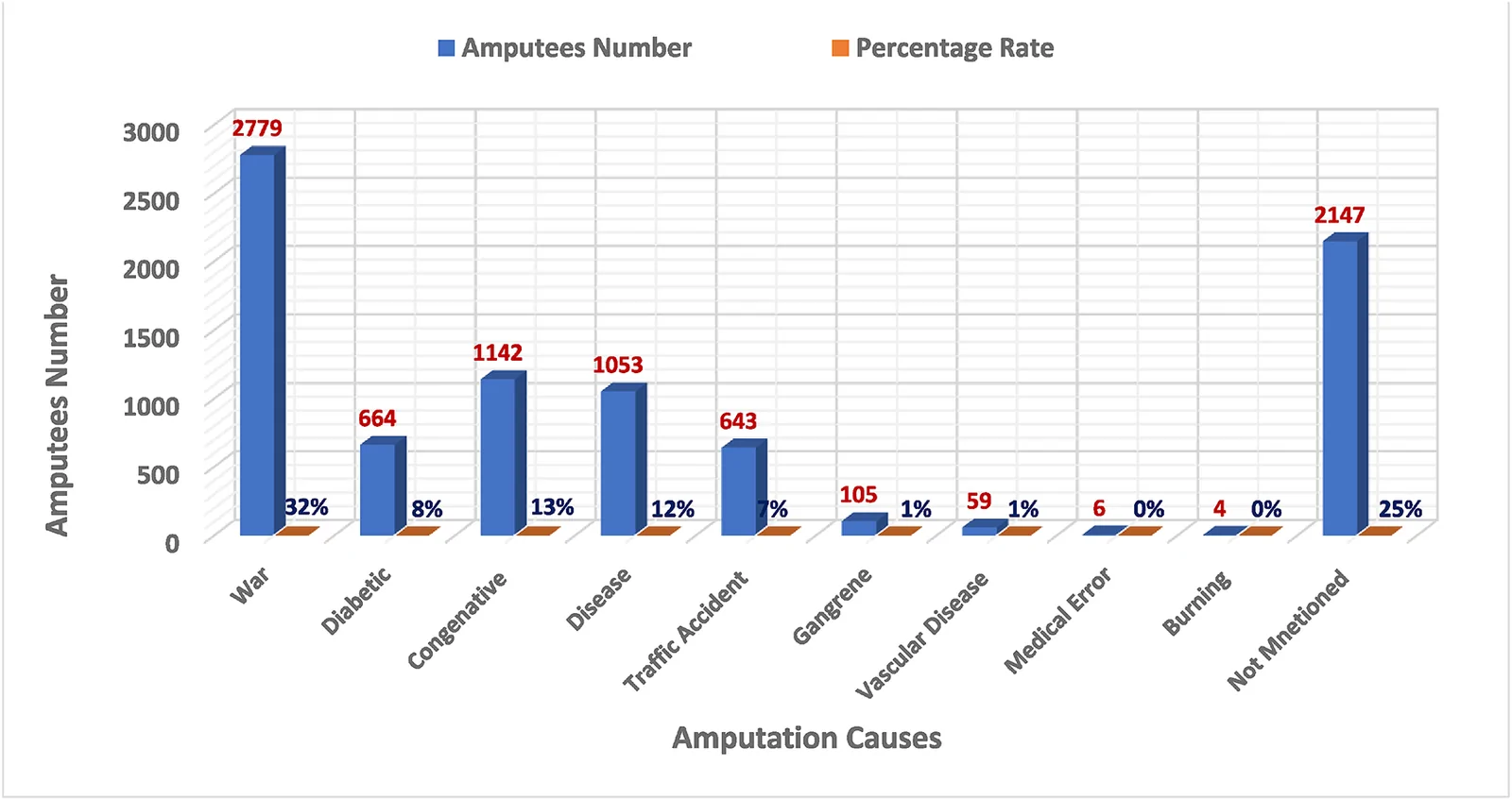
The effect of causes of locomotor dysfunction on the prevalence of amputees in Iraq in the age group from 40 to 60 years.

Upon analysing the lifestyle of individuals who have undergone amputation, the dataset clearly demonstrates that lifestyle as a variable plays a key role in the increasing prevalence of amputees in Iraq. Conversely, adolescents often have a lower probability of encountering circumstances that result in injuries and amputations. Furthermore, they have a lower susceptibility to chronic health conditions. Among individuals aged ≥70, the occurrence of amputations is relatively low due to factors such as the smaller population size, reduced mobility, and limited participation in military activities. While mobility issues can still contribute to a significant number of amputations in this age group, their overall prevalence is lower compared to other factors.

Fig. 4 illustrates that Babylon, Karbala, and Najaf had a significant population of people who had suffered amputations. The data also indicate that amputees from other locations in Iraq often visited these 3 clinics, potentially straining their available resources. The integration of rehabilitation, prosthetic, and orthotic centres has a significant impact on the distribution of prosthetic services, ensuring a fair allocation of amputees among the facilities.

**Fig. 4:**
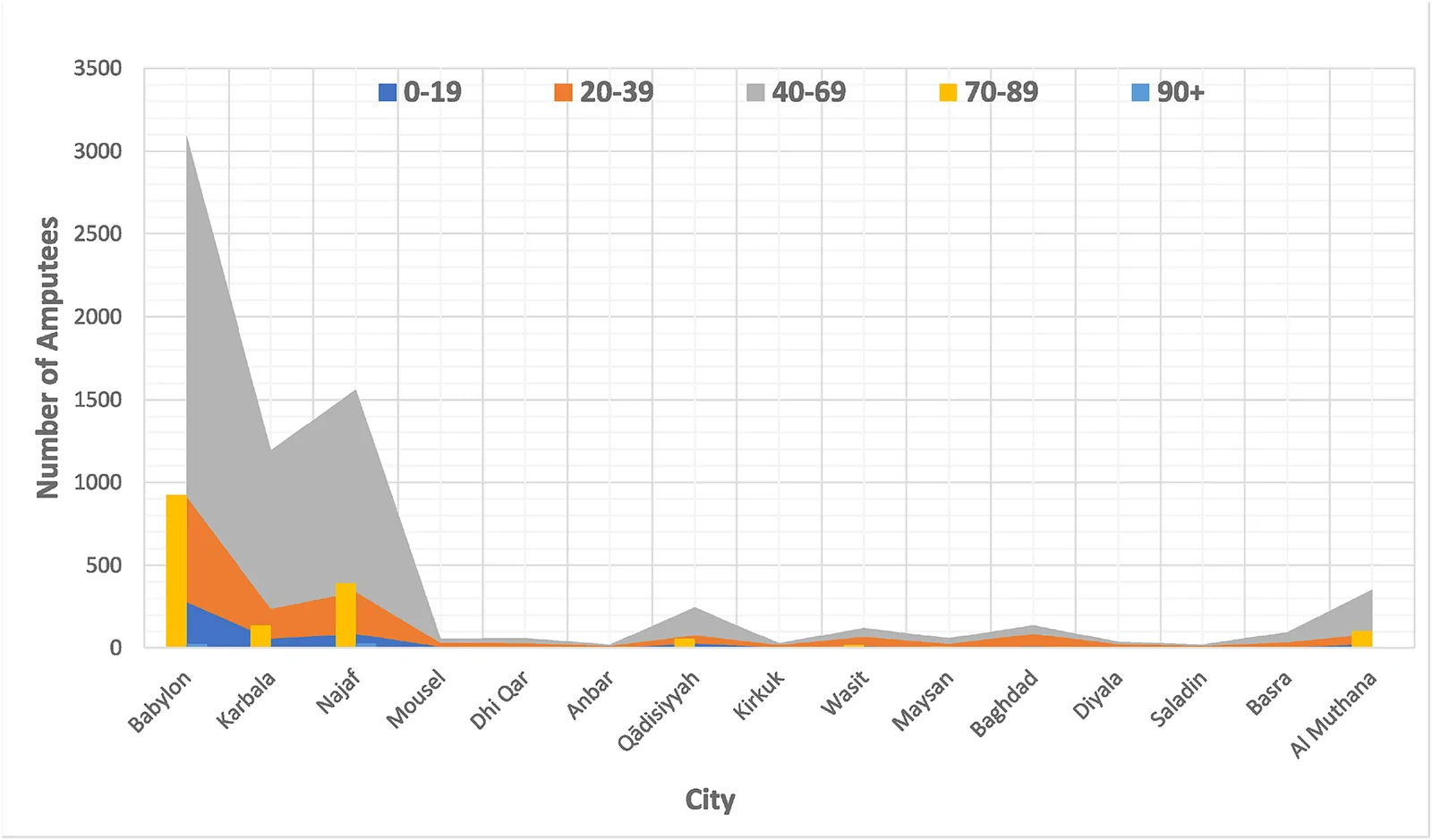
The distribution of amputees across different cities in Iraq.

According to Fig. 5, below-knee amputation (BK) was the most frequent type, accounting for a significant proportion of all amputations. Out of the 9030 amputees examined in the research, 5964 people suffered amputations below the knee, as shown in Fig. 5. The main factors contributing to this amount of amputation were warfare and diabetes. In addition, BKs provide superior prosthetic adaptation compared to amputations at other levels because of the anatomical form and optimal weight distribution. Moreover, the number of amputations at other levels was relatively low.

**Fig. 5:**
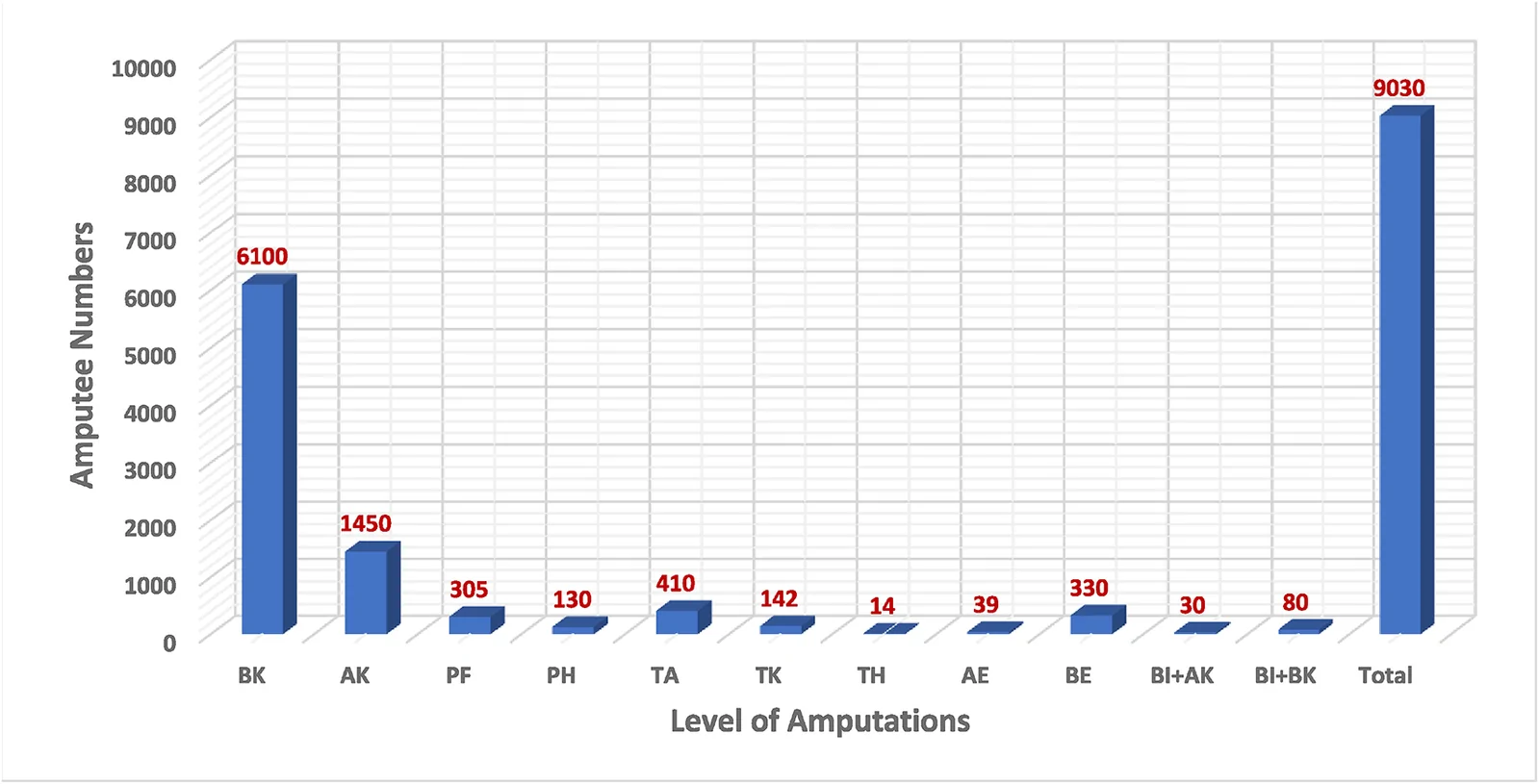
Prevalence of different levels of amputation among the amputees included in the research. BK: below-knee amputation; AK: above knee; PF: partial foot; PH: partial hand; TA: through ankle; TK: through knee; TH: through hip; AE: above elbow; BE: below elbow; BI: bilateral.

The years of conflict and war have resulted in a significant number of individuals losing their limbs. Fig. 6, Fig. 7 provide a clear visual representation of the notable influence of historical events, namely wars, on the frequency of amputations. The period from 1980 to 1989, characterised by the Iran-Iraq war, led to a significant number of casualties who suffered limb loss. Furthermore, the period between 2010 and 2019 coincides with significant hostilities in Iraq, such as the conflict with the United States and the emancipation of many cities from ISIS. These events likely resulted in a higher occurrence of conflict-related injuries and amputations, particularly BKs. The correlation between conflict and health outcomes emphasizes the significant impact of both war and diabetes on public health. It also highlights the ongoing need for rehabilitation programs and robust support networks for all those affected.

**Fig. 6:**
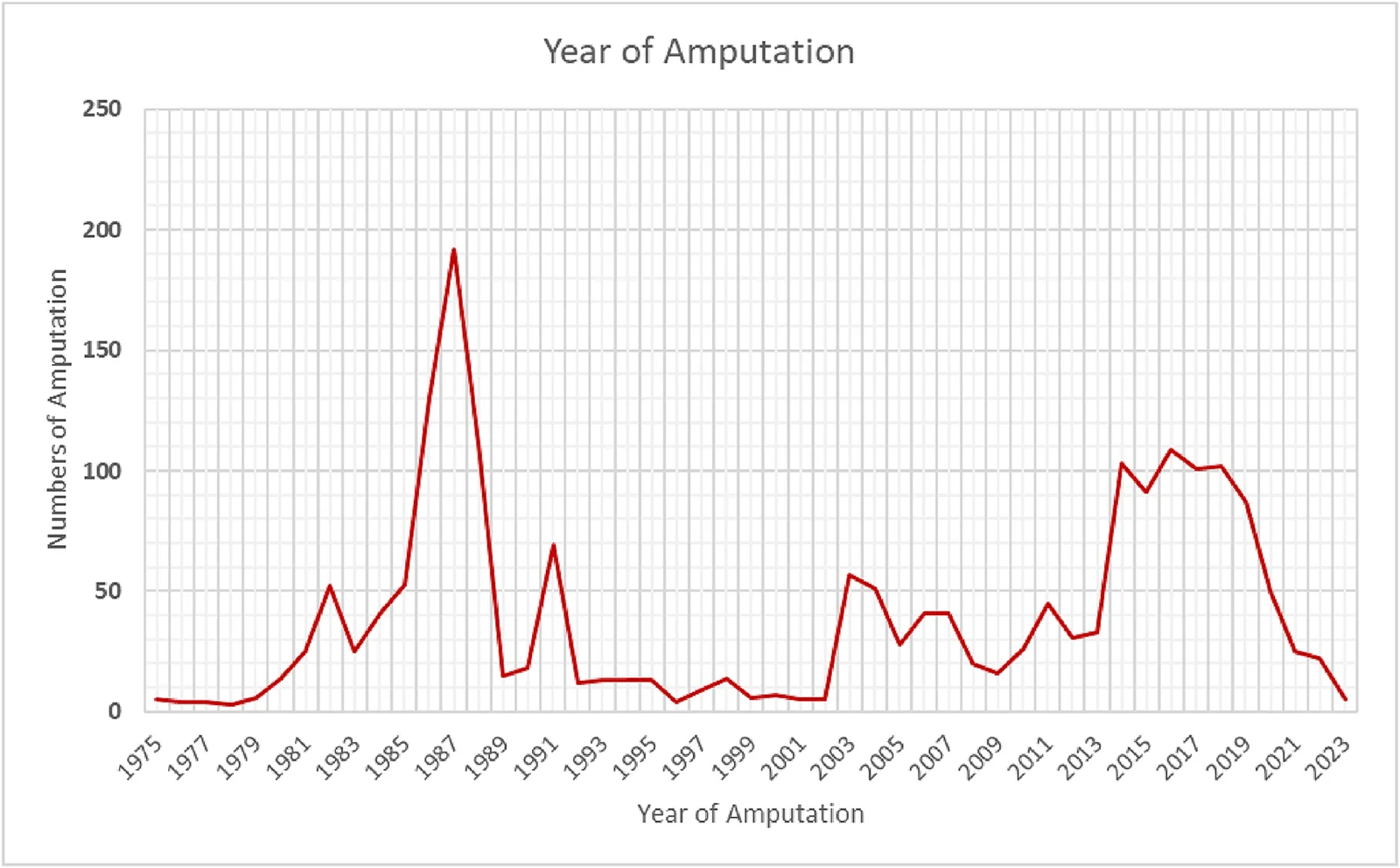
The temporal trend curve depicts the impact of amputee prevalences in 4 Iraqi cities.

**Fig. 7:**
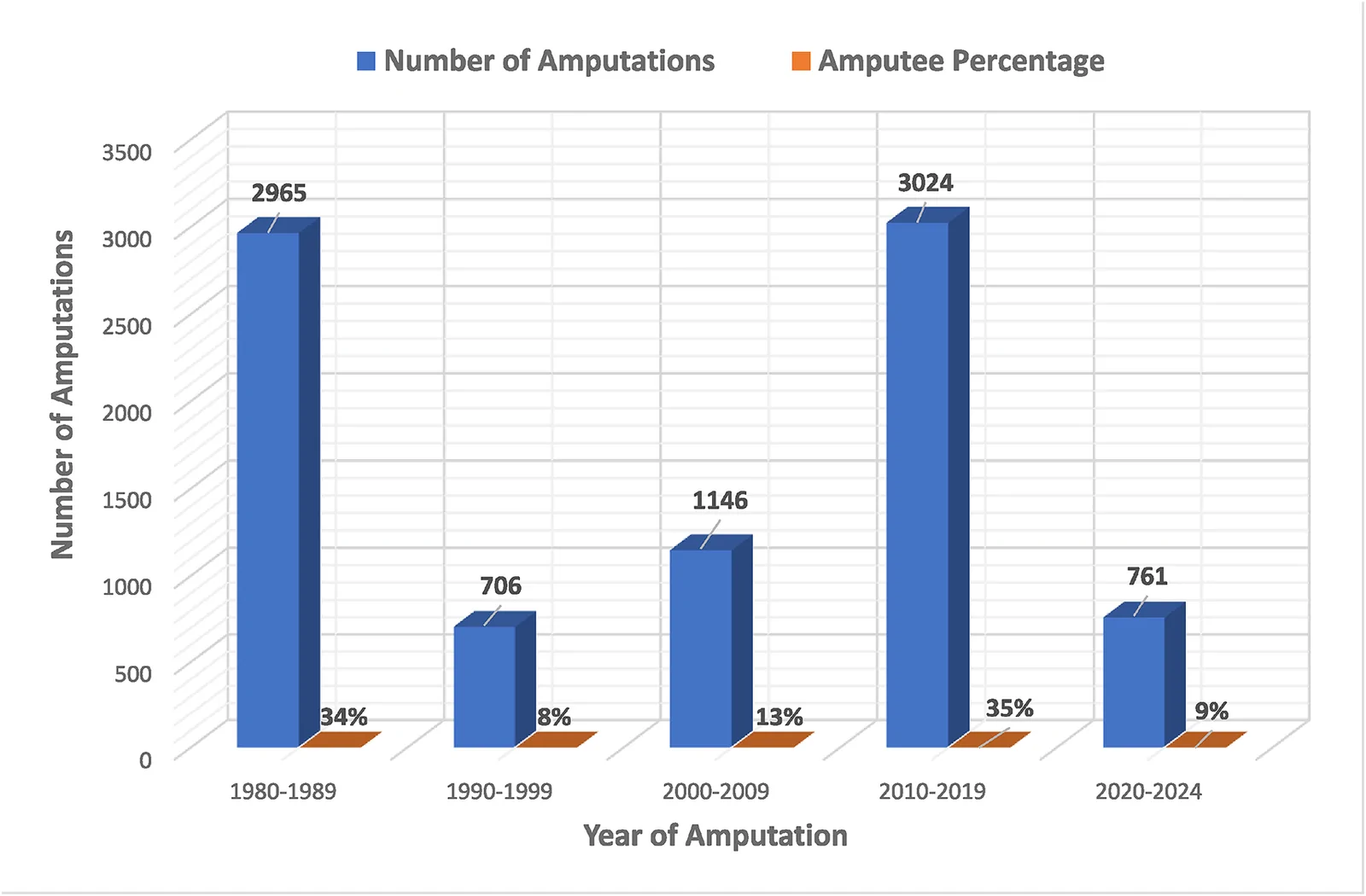
The correlation between the frequency of amputees and the passage of time.

The frequency of amputees among men and women is depicted in Fig. 8. Amputations have a greater impact on males. Occupation and exposure are the primary variables that contribute to the elevated number of male amputees. The Iraqi military is predominantly male, with approximately 99% males and 1% females. Fig. 8 illustrates the significant impact of war on this gender disparity. Moreover, there is a lower likelihood of women engaging in construction vocations in Iraq as compared to men. Furthermore, for 25% of amputees, the dataset did not include information on the reasons for amputation. This missing data is a crucial element that might impact the statistical analysis as well as the rehabilitation and prosthetic fitting procedures. The analysis of missing data in the Iraq centre record system is complicated because of the lack of precise information on the medical history of amputations.

**Fig. 8:**
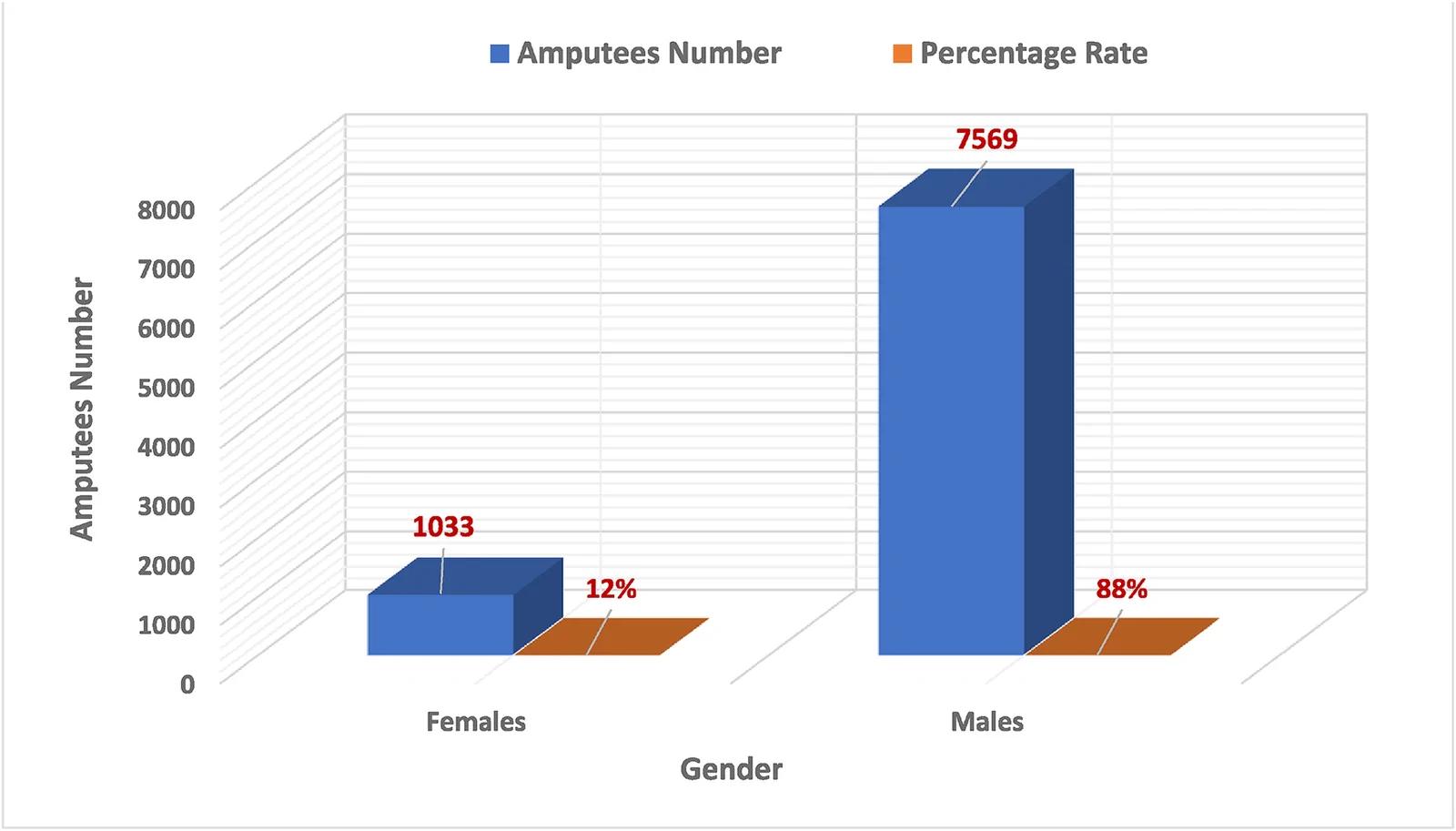
The incidence of amputation is much greater among male individuals compared to females.

Fig. 10 illustrates a significant gender disparity in amputation rates in Iraq, with men being far more affected than women. According to the statistics, 7410 males suffered lower-limb loss, while 1308 females had amputations for different causes. This discrepancy is primarily driven by factors such as war and job-related risks, to which men are disproportionately exposed.

Fig. 11 demonstrates that Iraq has the highest number of amputees compared to other countries included in the investigation. The Iraqi government did not have accurate statistics to document the prevalence of amputees or calculate the yearly rate of rise for each decade. The investigations conducted in the United States have also identified a significant number of amputees in the country resulting from both war-related injuries and diabetes. There was an enormous rise in the number of amputees due to war-related injuries and diabetes in Iraq and the United States compared to other nations.

Unlike the general global trend, the amputee prevalence is showing a substantial increase compared with other countries, even with the 4 states included in the study, rising from 102 to 152 per 100,000 individuals. The increase of about 49% indicated the worsening risk factors and serious gap in the healthcare system in the countries over time. The main potential influences were the war and disease. Unlike Iraq, the United States shows a significant reduction in the number of amputees, from 263 to 188 per 100,000, a drop of 28.5%, whereas Italy shows an improvement of about 30.7%.

## 4 Discussion

Meltable imputation methods were used to identify the missing data and its impact on the results. Table 1 and Fig. 12 show the results of the missing data that were collected from the health centres in the country. The Table shows that about 29.9% of the total number were missed, which could directly influence the rehabilitation process (Table 2).

**Table 1:** The total number of amputees that were not included because of the lack of documentation in the health center in Iraq.

Patient characteristics	Missing, *n* (%)	Valid, *n* (%)	Mean (SD)
Age	2700 (29.9)	6329 (70.1)	54.09 (18.166)
Cause of amputation	2507 (27.8)	6522 (72.2)	2.67 (2.409)
Amputation level	97 (1.1)	8932 (98.9)	1.88 (1.788)
Gender	2 (0)	9027 (99.98)	1.18 (0.380)

SD: standard deviation.

**Table 2:** The imputation models that were used to fill in missing data for 4 variables: age, cause of amputation, and amputation level.

Imputation models				
Patient characteristics	Model		Missing values	Imputed values
	Type	Effects		
Gender	Linear regression	Amputation level, cause of amputation, age	2	10
Amputation level	Linear regression	Gender, cause of amputation, age	97	485
Cause of amputation	Linear regression	Gender, amputation level, age	2507	12535
Age	Linear regression	Gender, amputation level, cause of amputation	2700	13500

Fig. 12 shows 9 distinct patterns of missingness. Red cells indicate missing values, while white cells represent observed data. The different patterns suggest that the missingness is not completely at random. It could also be missing at random, which is good for multiple imputation but requires careful model specification. Variables such as gender, which are mostly present but missing in some patterns (Fig. 8, Fig. 9), can serve as strong predictors when imputing other variables, if they are strongly correlated with them. The heavy reliance on multiple variables improves the quality of imputation but necessitates careful oversight to prevent overfitting, especially in models distinguished by numerous parameters (as illustrated by cause of amputation and age).

**Fig. 9:**
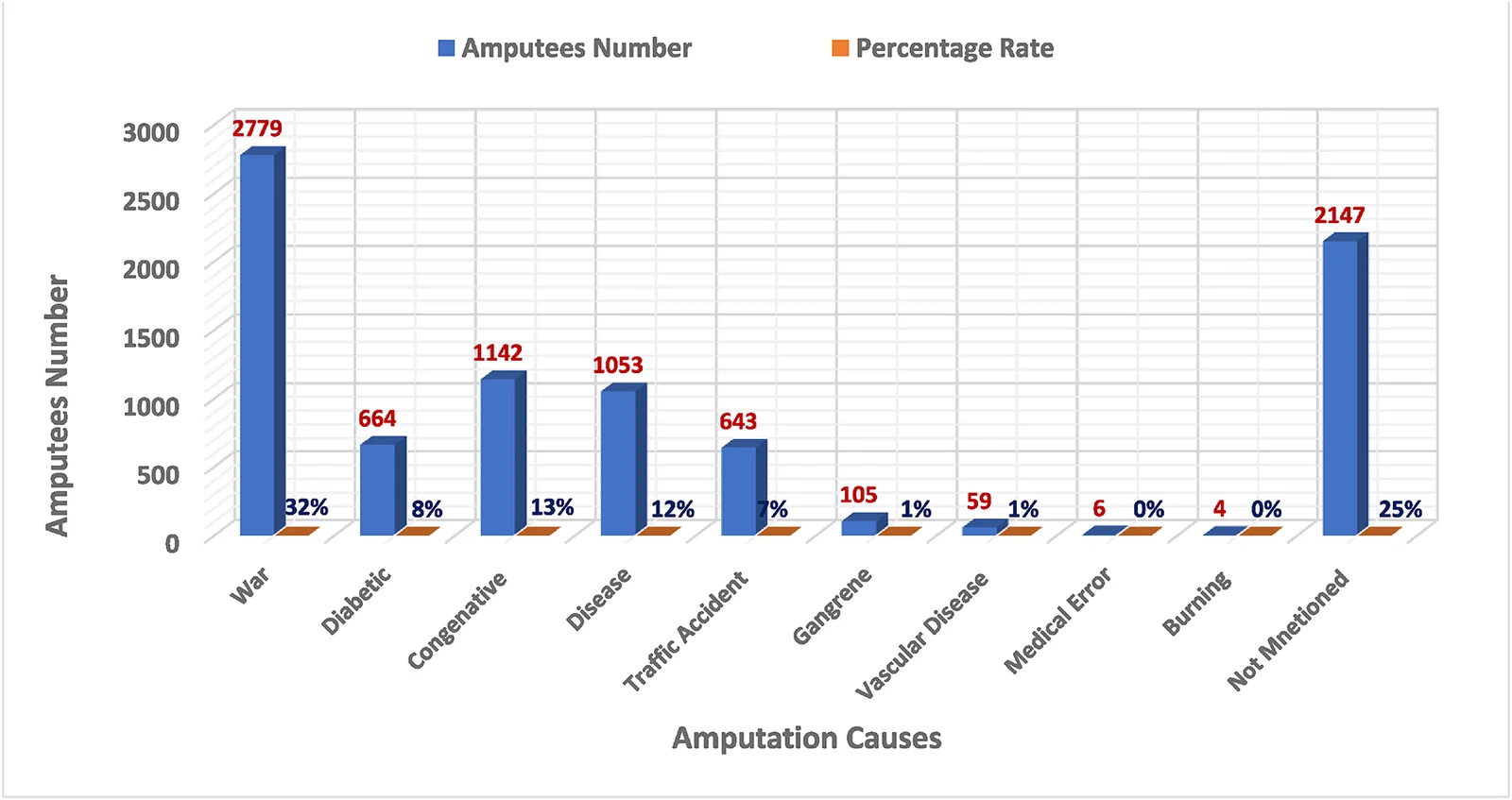
The correlation between the cause of amputation and the quantity of amputations in Iraq.

**Fig. 10:**
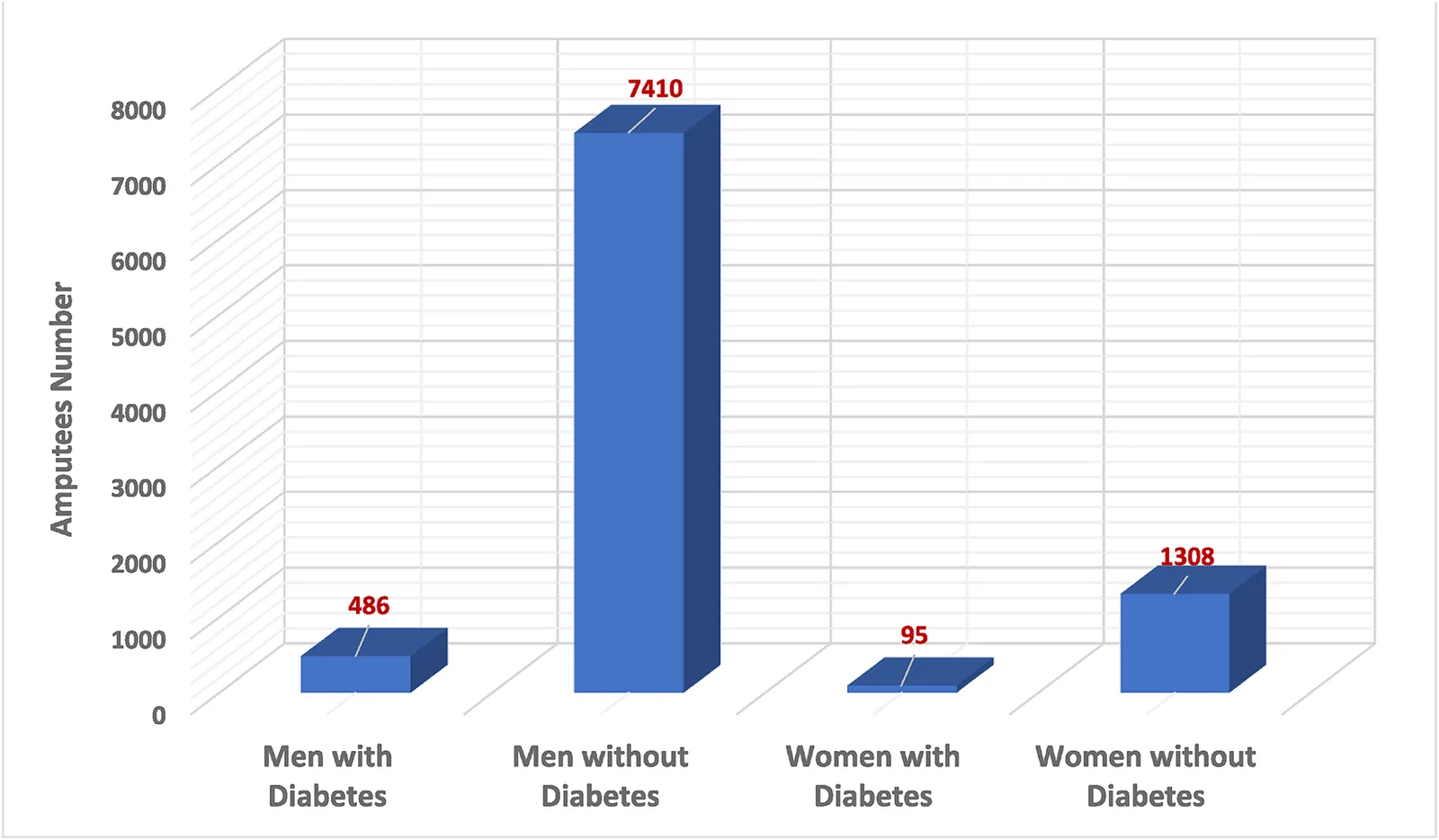
The impact of diabetes on the number of amputated individuals in 4 cities in Iraq.

**Fig. 11:**
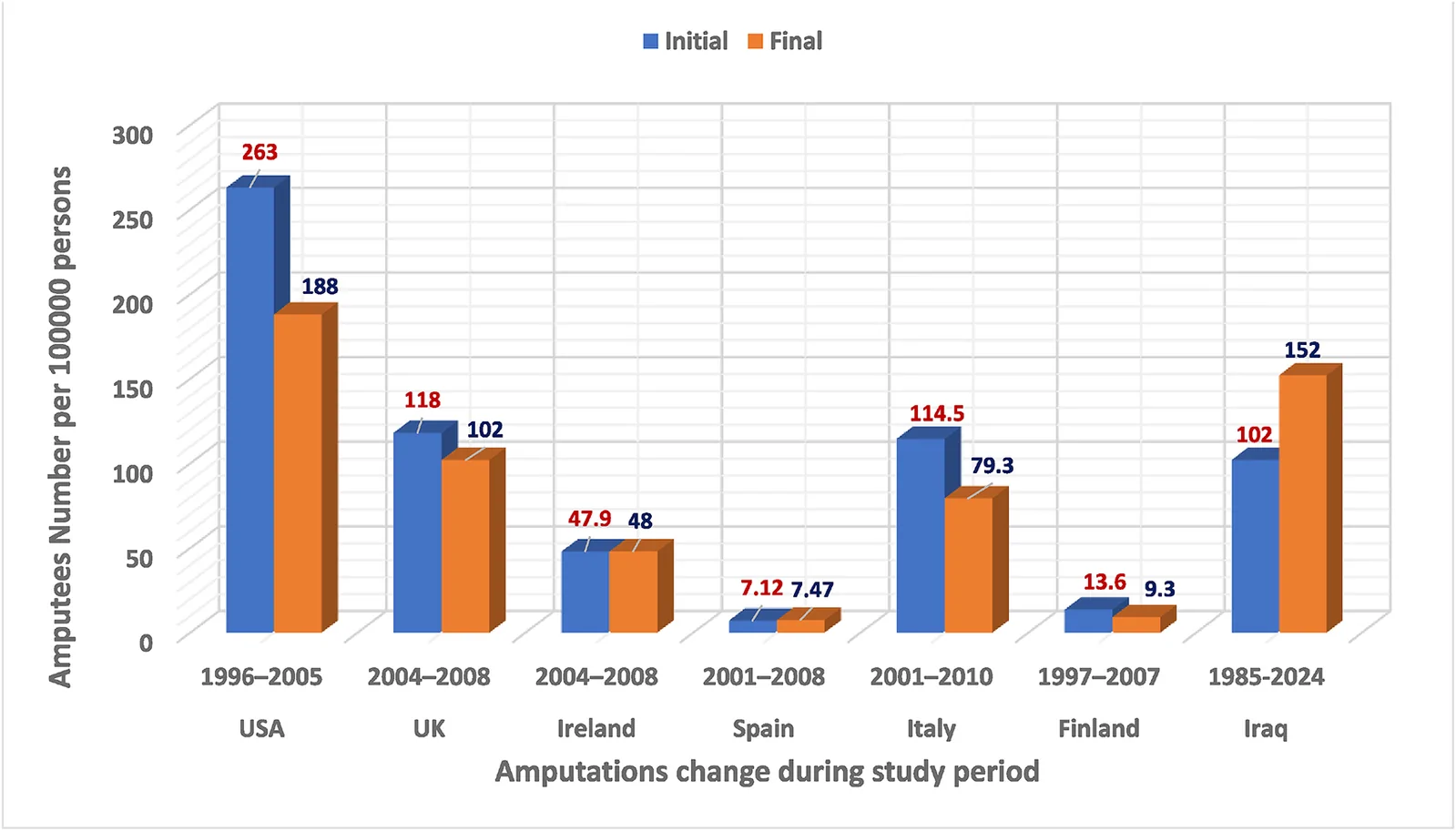
The variation in the prevalence of amputees in several nations, including Iraq, during a certain range of years. Each country's data is based on a variety of studies.^24^, ^25^, ^26^, ^27^, ^28^, ^29^

**Fig. 12:**
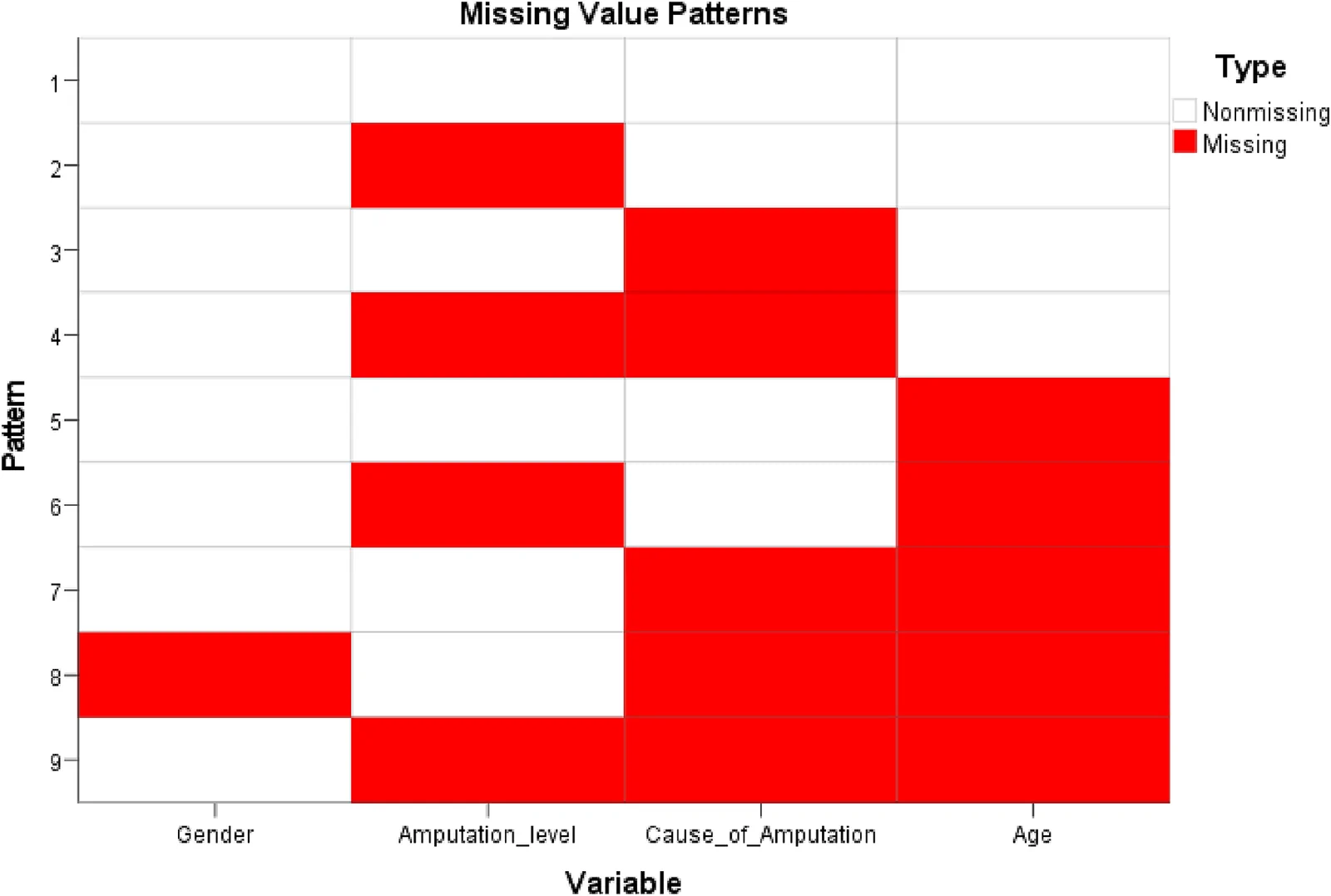
The missing data is spread out across 4 main variables: gender, amputation level, cause of amputation and age.

This study shows that multiple imputation provided stable group estimates, especially for the male group which had fewer missing values. The female group shows slightly more volatility and less efficiency due to a higher percentage of missing data. However, the final aggregated statistics are still reliable. The combination of low fraction missing Info. and high relative efficiency in both groups, as shown in Table 3, proves that the imputation method is valid and should be used instead of listwise deletion, which would have reduced the sample size and statistical power.

**Table 3:** Group statistics for the cause of amputation variable, broken down by gender (male = 1, female = 2).

Imputation number		Gender	No.	Mean (SD)	Std. error mean	Fraction missing Info.	Relative increase variance	Relative efficiency
Original data	Cause of amputation							
1	War	1	5421	2.50 (2.406)	0.033			
		2	1101	3.51 (2.242)	0.068			
2	Diabetic	1	7445	2.49 (2.396)	0.028			
		2	1582	3.44 (2.279)	0.057			
3	Traffic accident	1	7445	2.49 (2.411)	0.028			
		2	1582	3.48 (2.290)	0.058			
4	Gangrene	1	7445	2.51 (2.412)	0.028			
		2	1582	3.54 (2.286)	0.057			
5	Congenital	1	7445	2.51 (2.413)	0.028			
		2	1582	3.47 (2.249)	0.057			
6	Vascular disease	1	7445	2.51 (2.406)	0.028			
		2	1582	3.48 (2.324)	0.058			
Pooled	Medical error	1	7445	2.50 (−)	0.030	0.150	0.165	0.971
		2	1582	3.48 (−)	0.070	0.353	0.471	0.934

SD: standard deviation.

Limbs amputations are common surgical procedures for life-saving and curative purposes. Many causes of amputations among patients at the beginning of the census period are war-related injuries, congenital conditions, diseases, diabetes, and traffic accidents. The increase in the prevalence of amputations in Iraq from 1980 to 2024 reflects several factors and challenges that affected the country during that time. In the 1980s, Iraq experienced prolonged military conflict and domestic turmoil, leading to a rise in injuries that often resulted in limb loss through amputations.

In this study, the BKs recorded the highest number of amputations among other levels. The percentage of amputations increased after patients aged 40 to 70 years. Females are fewer in number than males. Additionally, from 1980 to 1994 and 2010 to 2020, armed conflicts and acts of terrorism exacerbated the existing security, economic, and social challenges in Iraq. The complex circumstances led to an increase in violent acts and injuries, resulting in an increased number of amputees in Iraq.

According to the research, Iraq is one of the countries with a high number of amputees compared to other countries. Reductions in healthcare funding have significantly impacted on Iraq's health services, impairing the system's capacity to provide essential treatment for amputees and establish a robust rehabilitation system. The large number of visitors to prosthetics, orthotics, and rehabilitation centres creates significant pressure on the facilities, affecting the quality of services and increasing the waiting period for providing prostheses to patients.

The medical records contained documentation errors and reporting discrepancies, resulting in missing data. Therefore, it needs to use statistical imputation methods to accurately determine the error rate. Imputation models were employed to meticulously analyze the study variables; however, their assumptions and predictive validity necessitate cautious interpretation. Although there are a lot of health centres in the country, the author could only get data from 4 different local states. This is because there is no centralized data collection system, and the authors must go there in person to collect the data.

Marked differences in lower extremity amputation rates across Iraq, Europe, North America, and East Asia have been previously documented by the Global Lower Extremity Amputation Study Group.^8^ These variations underscore the broader clinical relevance of understanding lower-limb biomechanics. Because the tibia functions as the principal weight-bearing bone during walking, examining how stresses and strains are distributed throughout the gait cycle is particularly important. In this context, finite element modelling offers a valuable approach for analysing stress concentration and deformation patterns within the lower and upper exoskeleton boons, such as tibia, femur, and foot.^30^

To address these challenges, it is crucial for the Iraqi populace, particularly those responsible for assisting amputees, to cooperate using the following approach:

(1) Creating a comprehensive national system for collecting data that precisely determines the number of amputees in Iraq and offers thorough patient histories, including information such as age, causes of amputation, level of amputation, gender, etc.
(2) It is of utmost importance to expand the number of prosthetics and orthotics health centres in cities across Iraq to alleviate the number of amputees on existing centres and ensure that patients receive the necessary therapy for their rehabilitation.
(3) Enhance patient awareness of ways to protect themselves against chronic illnesses, such as diabetes and gangrene, as well as potential post-surgery amputation-related complications.
(4) Raising awareness of safety and accident prevention, combined with rehabilitation and assistance initiatives, would facilitate their effective reintegration of amputees into society.
(5) The number of amputees in Iraq is significantly high due to many factors, specifically the wars and congenital issues, and disease as illustrated in the study. Implementing national-level initiatives for early diagnosis and management of risk factors such as diabetes, vascular disorders, and traumatic injuries can reduce the incidence of amputation. If individuals in Iraq learn about foot care and how to control diabetes avoiding injuries, they might be able to take better care of themselves. Improved post-operative care and standardised clinical pathways can facilitate prompt intervention. By keeping track of the reasons, treatments, and results of amputations, a national amputation registry can help shape public health policies.
(6) Evaluation of the impact of prevention and rehabilitation programs on amputation outcomes with the help of the World Health Organization.

## CRediT authorship contribution statement

**Amer Matrood Imran:** Writing – review & editing, Writing – original draft, Software, Methodology, Formal analysis, Data curation. **Adnan K. Shathir:** Software, Data curation.

## Ethical statement

This study was conducted using retrospective anonymized data obtained with official approval from the participating health centres. No personal identifiers, including patient names, were collected or accessed during the study. As the data were fully anonymized, informed consent was not required in accordance with local institutional regulations.

## Funding

The authors received no financial support for the research, authorship, and/or publication of this article.

## Declaration of competing interest

The authors declare that they have no known competing financial interests or personal relationships that could have appeared to influence the work reported in this paper.

## References

[1] Omotayo O., Maduka C.P., Muonde M. (2024). The rise of non-communicable diseases: a global health review of challenges and opportunities. Int Med Sci Res J.

[2] Imran A., Beigzadeh B., Haghjoo M.R. (2023). A new passive transfemoral prosthesis mechanism based on 3R36 knee and ESAR foot providing walking and squatting. Theor Appl Mech Lett.

[3] McDonald C.L., Westcott-McCoy S., Weaver M.R. (2021). Global prevalence of traumatic non-fatal limb amputation. Prosthet Orthot Int.

[4] Kröger K., Berg C., Santosa F. (2017). Lower limb amputation in Germany. Dtsch Arztebl Int.

[5] Sun T., Liu S., Xie F. (2021). Influence of road types on road traffic accidents in northern Guizhou province, China. Chin J Traumatol (Engl Ed).

[6] Sun T.J., Huang X.F., Xie F.K. (2025). Road traffic mortality in zunyi city, China: a 10 - year data analysis (2013-2022). Chin J Traumatol (Engl Ed).

[7] Krueger C.A., Wenke J.C., Ficke J.R. (2012). Ten years at war: comprehensive analysis of amputation trends. J Trauma Acute Care Surg.

[8] Unwin N. (2000). Epidemiology of lower extremity amputation in centres in Europe, North America and East Asia. Br J Surg.

[9] Kaeppler EPv, Hetherington A., Donnelley C.A. (2021). Impact of prostheses on quality of life and functional status of transfemoral amputees in Tanzania. Afr J Disabil.

[10] Davie-Smith F., Paul L., Nicholls N. (2017). The impact of gender, level of amputation and diabetes on prosthetic fit rates following major lower extremity amputation. Prosthet Orthot Int.

[11] Samuelsen B.T., Andrews K.L., Houdek M.T. (2017). The impact of the immediate postoperative prosthesis on patient mobility and quality of life after transtibial amputation. Am J Phys Med Rehabil.

[12] Kamitani F., Nishioka Y., Noda T. (2021). Incidence of lower limb amputation in people with and without diabetes: a nationwide 5-year cohort study in Japan. BMJ Open.

[13] Zhang Y., van Netten J.J., Baba M. (2021). Diabetes-related foot disease in Australia: a systematic review of the prevalence and incidence of risk factors, disease and amputation in Australian populations. J Foot Ankle Res.

[14] Alvarsson A., Sandgren B., Wendel C. (2012). A retrospective analysis of amputation rates in diabetic patients: can lower extremity amputations be further prevented. Cardiovasc Diabetol.

[15] Malyar N.M., Freisinger E., Meyborg M. (2016). Low rates of revascularization and high In-Hospital mortality in patients with ischemic lower limb amputation: morbidity and mortality of ischemic amputation. Angiology.

[16] Richardson C., Glenn S., Nurmikko T. (2006). Incidence of phantom phenomena including phantom limb pain 6 months after major lower limb amputation in patients with peripheral vascular disease. Clin J Pain.

[17] Khalid W., Yasmeen H., Raza A. (2025). Prevalence of phantom pain in patients with lower limb amputation in District Swabi. Heal J Physiother Rehabil Sci.

[18] Yilmaz M., Gulabi D., Kaya I. (2016). The effect of amputation level and age on outcome: an analysis of 135 amputees. Eur J Orthop Surg Traumatol.

[19] Li W.S., Chan S.Y., Chau W.W. (2019). Mobility, prosthesis use and health-related quality of life of bilateral lower limb amputees from the 2008 Sichuan earthquake. Prosthet Orthot Int.

[20] Stergiou A., Tzoufi M., Ntzani E. (2017). Therapeutic effects of horseback riding interventions: a systematic review and meta-analysis. Am J Phys Med Rehabil.

[21] Donnelley C.A., Shirley C., Epv Kaeppler (2021). Cost analyses of prosthetic devices: a systematic review. Arch Phys Med Rehabil.

[22] Daigeler A., Lehnhardt M., Khadra A. (2009). Proximal major limb amputations - a retrospective analysis of 45 oncological cases. World J Surg Oncol.

[23] Sun J.H., Tsai J.S., Huang C.H. (2012). Risk factors for lower extremity amputation in diabetic foot disease categorized by Wagner classification. Diabetes Res Clin Pract.

[24] Goodney P.P., Beck A.W., Nagle J. (2009). National trends in lower extremity bypass surgery, endovascular interventions, and major amputations. J Vasc Surg.

[25] Vamos E.P., Bottle A., Edmonds M.E. (2010). Changes in the incidence of lower extremity amputations in individuals with and without diabetes in England between 2004 and 2008. Diabetes Care.

[26] Buckley C.M., O'Farrell A., Canavan R.J. (2012). Trends in the incidence of lower extremity amputations in people with and without diabetes over a five-year period in the Republic of Ireland. PLoS One.

[27] Lopez-de-Andres A., Jiménez-García R., Aragón-Sánchez J. (2015). National trends in incidence and outcomes in lower extremity amputations in people with and without diabetes in Spain, 2001-2012. Diabetes Res Clin Pract.

[28] Lombardo F.L., Maggini M., De Bellis A. (2014). Lower extremity amputations in persons with and without diabetes in Italy: 2001-2010. PLoS One.

[29] Winell K., Venermo M., Ikonen T. (2013). Indicators for comparing the incidence of diabetic amputations: a nationwide population-based register study. Eur J Vasc Endovasc Surg.

[30] Alsaeedi S., Mohsen A.M.H., Nile K.K. (2024). Inductive 3D numerical modelling of the tibia bone using MRI to examine von Mises stress and overall deformation. Open Eng.

